# Comparison of novel oral anticoagulants versus warfarin for post-operative atrial fibrillation after coronary artery bypass grafting

**DOI:** 10.1016/j.amsu.2020.09.007

**Published:** 2020-09-08

**Authors:** Lucy Manuel, Laura S. Fong, Zhen Hao Ang, Peter Grant

**Affiliations:** Department of Cardiothoracic Surgery, Prince of Wales Hospital, Sydney, Australia

**Keywords:** Review, Coronary artery bypass, Novel oral anticoagulant, Warfarin, Atrial fibrillation

## Abstract

A best evidence topic in cardiac surgery was written according to a structured protocol. The question addressed was ‘Does the use of Novel Oral Anticoagulants (NOACs) result in more complications than Warfarin for treatment of post-operative atrial fibrillation (AF) following coronary artery bypass grafting (CABG)?’ Altogether more than 93 papers were found using the reported search with 4 studies representing the best evidence to answer the clinical question, including 1 randomised trial and 3 retrospective case-control studies. The authors, journal, date and country of publication, patient group studied, study type, relevant outcomes and results of these papers were tabulated. Timing for initiation of anticoagulation was similar across the studies, with both demonstrating longer hospital stays and greater time to reach therapeutic anticoagulation in the warfarin cohort. Three studies reported similar safety between the two groups. One study revealed significantly more invasive interventions for pleural or pericardial effusions in the NOAC group, whilst in contrast another study demonstrated a higher rate of major bleeding in the warfarin cohort. Cost-analysis revealed that NOACs were overall more cost-effective compared to warfarin despite the higher cost for the medication itself. In conclusion, the use of NOACs after CABG for post-operative AF can be used as an alternative to warfarin, however, one should remain vigilant for possible pericardial or pleural effusions which may require reintervention. Further dedicated research and larger appropriately powered randomised control trials are needed to confirm the safety of NOACs in post-cardiac surgery patients.

## Highlights

• Atrial fibrillation (AF) is the most common arrhythmia post cardiac surgery.
• While usually self-limiting, AF has increased morbidity and mortality to patients.
• Anticoagulation is warranted if AF is persistent or recurrent.
• Warfarin has been the mainstay of therapy, however, NOACs have gained popularity.
• The safety of NOACs in post-cardiac surgery patients is so far poorly defined.

## 1 Introduction

A best evidence topic was constructed according to a structured protocol. This is fully described in a previous publication in the IJS [1].

## 2 Clinical scenario

A 74-year-old man develops new atrial fibrillation (AF) four days following CABG. The patient's family report that they have heard of new blood thinning agents where daily blood tests and monitoring of “levels” is not required. Although you are aware that NOACs have been approved for the use of non-valvular AF and the prevention and treatment of deep vein thrombosis, you are concerned about commencing a NOAC in a high-risk post-cardiac surgery patient. You resolve to review the literature to determine the safety of NOAC use for AF in the post-operative CABG patient.

## 3 Three-part question

In patients following coronary artery bypass grafting (CABG) with atrial fibrillation (AF) does the use of novel oral anticoagulants (NOACs) result in more complications than warfarin?

## 4 Search strategy

A literature search was performed on the MEDLINE database (1964 to present) using the OVID interface with the terms ‘coronary artery bypass’ [all fields] AND ‘Direct Oral Anticoagulant’ [all fields] OR ‘warfarin’ [all fields] OR ‘NOAC’ [all fields] OR ‘Novel Oral Anticoagulant’ [all fields]. The reference lists of initially identified papers were searched for other relevant studies. The search was current as of August 5, 2020.

## 5 Search outcome

A total of 93 papers were found using the reported search. From these 9 were not in English, 55 were irrelevant, 13 were case reports or editorial commentary, 7 focused on single agent anticoagulation or antiplatelet medication, 3 were post-angioplasty, and 2 related to valvular procedures. The remaining 4 papers directly compared warfarin and NOAC administration for post-operative CABG patients with AF and were therefore chosen as the best evidence to answer the clinical question.

## 6 Results

The results of the four papers (three retrospective case-control studies and one randomised control trial) are summarised in Table 1.

**Table 1:** Best evidence papers.

Author, date, journal and country, study type (level of evidence)	Patient group	Outcomes	Key results NOAC vs. Warfarin	Comments
Yu et al., 2019, Journal of Cardiac Surgery, US [2] Case-control study (level III)	246 patients NOAC: 64 Warfarin: 182	Post-operative day for initiating anticoagulation	4.0 vs. 3.0 (p < 0.001)	This study demonstrated that patients commenced on NOACs had significantly more effusions requiring invasive interventions compared to those receiving warfarin. Additionally, those on NOACs were more likely to require delayed intervention.
		Hospital length of stay (days)	8.0 vs. 9.0 (p < 0.003)	
		Invasive intervention for pericardial and pleural effusions (total)	26.6% vs. 13.2% (p < 0.014)	
		No intervention	73.4% vs. 86.8% (p < 0.001)	
		Early effusion (presenting within index admission)	4.7% vs. 9.3% (p = NR)	
		Late effusion (up to 3 months following discharge)	21.9% vs. 3.8% (p < 0.001)	
Anderson et al., 2015, American Journal of Surgery, US [3] Case-control study (level III)	167 patients- new post-operative AF 72 patients treated with anticoagulation NOAC: 27 Warfarin: 45	Post-operative day for initiating anticoagulation	4.3 vs. 3.9 (p = NS)	This study demonstrated no significant differences in length of stay or blood product usage between the two groups. However, NOACs were more cost-effective than warfarin, when the price of the medication and related blood tests was considered.
		Post-operative length of stay (days)	6.6 vs. 7.3 (p = NS)	
		Time to reach therapeutic anticoagulation (days)	4.3 vs. 8.4 (p < 0.001)	
		Use of blood products post anticoagulation	0.7 vs. 0 (p = NS)	
		Drug cost (US dollar)	377.92 vs. 20.04 (p < 0.001)	
		Mean total anticoagulant related cost (US dollar)	377.92 vs. 857.41 (p < 0.001)	

Woldendorp et al., 2020, Heart, Lung and Circulation, Australia [4] Case-control study (level III)	960 patients isolated CABG 305 post-operative AF NOAC: 29 Warfarin: 77	Post-operative length of stay (days)	12.4 ± 7.9 vs. 13.6 ± 7.8 (p = NS)	This study demonstrated no additional risk to patients in terms of early or late morbidity and mortality. Notably, patients started on a NOAC were less likely to be smokers or have a history of heart failure and were more likely to have had off-pump surgery.
		Pericardial effusion (patients)	0 vs. 1 (p = 0.55)	
		Major/minor bleeding	0 vs. 3 (p = 0.02)	
Chapin et al., 2020, Journal of Cardiovascular Pharmacology and Therapeutics, US [5] Randomised Control Trial (level II)	56 patients NOAC: 28 Warfarin: 28	Post-operative day of onset of AF	2.04 ± 1.35 vs. 2.18 ± 1.36 (p = 0.70)	Pilot study not adequately powered to determine a statistical significance in safety or efficacy. There was no difference in STS calculated mortality/morbidity scores between the two groups.
		ICU post-operative length of stay (days)	2.14 ± 1.41 vs. 1.43 ± 0.50 (p = 0.02)	
		Total post-operative length of stay (hours)	167.5 ± 76.6 vs. 143.1 ± 53.2 (p = 0.17)	
		Transfusions as inpatient	2 vs. 1 (p = 1.00)	
		Transfusions after discharge	1 vs. 0 (p = 1.00)	
		Thoracentesis for pleural effusion	8 vs. 6 (p = 0.65)	
		Epistaxis requiring intervention	2 vs. 0 (p = 0.49)	
		Epistaxis not requiring intervention	1 vs. 0 (p = 1.00)	
		GI bleed requiring endoscopy	1 vs. 0 (p = 1.00)	
		Number of days of inpatient anticoagulation	3.4 ± 3.1 vs. 2.3 ± 1.8 (p = 0.139)	
		Drug cost (US dollar)	9.80 ± 8.27 vs. 0.75 ± 0.43 (p < 0.001)	
		Total cost comparison (US dollar)	522.50 ± 91.40 vs. 778.22 ± 248.80 (p = 0.003)	

*AF,* atrial fibrillation; *CABG,* coronary artery bypass grafting; *NOAC,* novel oral anticoagulant; *STS*, Society of Thoracic Surgery.

## 7 Discussion

Post-operative AF is the most common arrhythmia following cardiac surgery, with an estimated incidence of 20–40% in isolated CABG procedures [4]. While usually self-limiting, AF increases morbidity and mortality, with an increased risk of stroke, prolonged hospitalisation and other thromboembolic complications [5,6]. When AF is persistent or recurrent, anticoagulation is indicated, with warfarin being the current mainstay of treatment. Use of anticoagulants in cardiac surgery patients requires special consideration, with greater risk of bleeding and cardiac tamponade in the post-operative period in this high-risk population [6]. In the last decade, NOACs have gained popularity and demonstrated safety for the treatment of non-valvular AF, however, their role remains controversial in post-operative cardiac surgery patients [4].

Yu et al. [2] completed a single-centre retrospective review in 2019 comparing 246 patients who were anticoagulated with either a NOAC or warfarin for post-operative AF following CABG. Records were reviewed from 2014 to 2017, with 64 (26%) patients receiving NOAC and 182 (74%) commencing warfarin. Despite patients being commenced on warfarin earlier than the NOAC cohort (Day 3.0 vs. 4.0, p < 0.001), this group had significantly longer hospital length of stays (9.0 vs. 8.0 days, p < 0.001), with 56.6% of warfarin patients being bridged with intravenous heparin infusion. Patients were significantly more likely to require invasive intervention (insertion of chest drain, thoracentesis, re-exploration for bleeding) when commenced on a NOAC (NOAC 26.6% vs. warfarin 13.2%, p < 0.0014). Multivariable log-binomial regression revealed that those patients receiving NOACs were 2.14 times more likely to develop effusions requiring interventions compared to warfarin (95% confidence interval 1.26–3.63, p < 0.005). Notably, although the value did not reach statistical significance, those in the warfarin cohort were twice as likely to develop an early effusion within the index admission (4.7% vs. 9.3% (p = NR)). The patients were followed for 3 months post-operatively where late effusion rates were significantly higher in the NOAC group (21.9% vs. 3.8%, p < 0.001).

Similarly, Anderson et al. [3] in 2015 performed a single-centre retrospective review of patients who developed post-operative AF following CABG between 2013 and 2015. A total of 598 patient records were reviewed, of which 184 developed post-operative AF and of these, 72 received anticoagulation as a component of their management. There was no significant difference in the post-operative day for commencement of anticoagulation in either the NOAC or warfarin group (4.3 vs. 3.9, p = NS) or total hospital length of stay (6.6 vs. 7.3, p = NS). The time taken to reach therapeutic anticoagulation levels was significantly longer in the warfarin cohort (8.4 vs. 4.3 days, p < 0.001). Within the warfarin cohort, 62% (28/45) of patients received bridging therapy- 27 patients with enoxaparin and 1 patient receiving unfractionated heparin. Neither group had bleeding complications in the index hospitalisation due to anticoagulation. One patient in the NOAC group required a transfusion for post-operative anaemia following initiation of therapy. Two patients in the warfarin cohort required readmission and reoperation for major bleeding complications including gastrointestinal bleeding and redo-sternotomy for pericardial tamponade. There were no late bleeding complications however, the length of long-term follow-up was unclear. A cost analysis demonstrated that warfarin was significantly more cost-effective when considering only the price of the medication (US$20.04 vs. US$377.92, p < 0.001). However, total costs including blood tests to monitor international normalised ratio (INR) and laboratory processing fees resulted in warfarin being significantly more expensive over 30 days with a mean cost of US$857.41 (vs. US$377.92, p < 0.001). Costs of reintervention when required were not included in the cost analysis.

Woldendorp et. Al [4], completed a single centre retrospective study in 2020 comparing management of patients who developed post-operative AF following isolated CABG. A total of 960 patients underwent an isolated CABG between January 2015 and December 2018, with 305 (31.8%) of patients developing post-operative AF. Of these, 106 were discharged on anticoagulation, 77 on warfarin and 29 on a NOAC. Anticoagulation was initiated in patients at the physician's discretion with a general guideline of episodes that lasted for >24 h or two or more shorter episodes of AF. Older patients were significantly more likely to develop post-operative AF (67.7 ± 9.4 years vs. 63.0 ± 10.7 years, p < 0.001), as well as those with a previous history of cerebrovascular disease (14.6% vs. 8.7%, p = 0.02) and a higher CHADS-VASc score (2.5 vs. 2.8, p < 0.001). Patients who developed AF were more likely to have early mortality (2.6% vs. 0.8%, p = 0.03), develop renal failure (4.9% vs. 1.8%, p = 0.02), and were more likely to return to theatre (16.7% vs. 9.9%, p = 0.002). Moreover, those patients who underwent off-pump surgery were less likely to develop post-operative AF (29.8% vs. 37.1%, p = 0.03). Hospital length of stay was increased in patients who developed AF (12.6 ± 10.6 vs. 9.3 ± 16.3, p < 0.001), however, the choice of anticoagulant did not significantly influence length of stay (12.4 ± 7.9 vs. 13.6 ± 7.8 (p = NS)). Significant bleeding events were not found to be increased in patients who were anticoagulated, regardless of the drug used. There were no readmissions for bleeding or pericardial effusions in the 1 year following surgery for patients discharged on NOACs. 4 patients were readmitted after being discharged on warfarin, 1 for a pericardial effusion (0 vs. 1 (p = 0.55)) and 3 for major bleeding (0 vs. 3 (p = 0.02)). All patients had recent evidence of a supratherapeutic INR.

Chapin et. Al [5], designed a randomised control pilot study comparing the efficacy, safety and cost of apixaban vs. warfarin for patients with post-operative AF following CABG. Patients who developed new onset post-operative AF were randomised from 2016 to 2019 with 56 being allocated to the trial. In order to be included in the study, patients had new onset post-operative AF, defined by AF on telemetry for >12 h or multiple episodes of AF each lasting longer than 30 min 11 patients (39.3%) on warfarin deemed to be high risk for thromboembolism received bridging therapy with enoxaparin. There was no in hospital or 30-day mortality, strokes or thromboembolic events in either group. Although bleeding complications occurred in both groups with similar frequency, no patients required reoperation. Pleural effusion requiring thoracentesis was the most common complication, however, the numbers were similar among the NOAC and warfarin group (8 vs. 6, p = 0.65). There was no significant difference in blood transfusions, epistaxis or GI bleeds (3 vs. 1 (p = NS); 3 vs. 0 (p = NS); (1 vs. 0 (p = NS)). Longer ICU length of stay among NOAC patients (2.14 ± 1.41 vs. 1.43 ± 0.50 (p = 0.02)) was hypothesised to relate to higher proportion of patients with myocardial infarction in apixaban group (17 vs. 9, p = 0.32). Despite this randomisation, there was no difference in STS mortality/morbidity scores between the two cohorts. As in previous studies [3], warfarin was significantly more cost effective when comparing the drug price alone (US$0.75 ± 0.43 vs. 9.80 ± 8.27 (p < 0.001), with apixaban having significantly lower total costs (US$522.50 ± 91.40 vs. 778.22 ± 248.80 (p = 0.003)). Total costs included the medication, INR laboratory monitoring, round-trip traveling to laboratory utilised by patient, and bridging when required.

## 8 Clinical bottom line

Anticoagulation following cardiac surgery requires special consideration due to the susceptibility of bleeding complications in this high-risk population which can cause severe hemodynamic compromise necessitating the need for urgent reintervention. Studies comparing NOACs to warfarin for post-operative AF following CABG are limited in number and quality- and is an area of current investigative interest. While there has been one randomised pilot trial, the numbers were limited and the study not sufficiency powered. Moreover, the available retrospective studies are restricted by their small cohort sizes and short lengths of follow-up. Only one study demonstrated a higher rate of reintervention and readmission for NOACs, while another study demonstrated a higher rate of major bleeding for warfarin. Cost-analysis demonstrates that NOACs overall are more cost-effective when compared to warfarin despite the higher cost for the medication itself. In conclusion, the use of NOACs after CABG for post-operative AF can be used as an alternative to warfarin, however, one should remain vigilant for possible pericardial or pleural effusions which may require reintervention. Further dedicated research and larger appropriately powered randomised control trials are needed to confirm the safety of NOACs in post-cardiac surgery patients.

## Sources of funding

Nil funding for research.

## Ethical approval

Nil ethics approval required.

## CRediT authorship contribution statement

**Lucy Manuel:** Formal analysis, Writing - original draft, Literature search, analysis and writing of manuscript. **Laura S. Fong:** Formal analysis, Writing - review & editing, Analysis and editing of manuscript. **Zhen Hao Ang:** Writing - review & editing, Formal analysis, Analysis and editing of manuscript. **Peter Grant:** Writing - review & editing, Formal analysis, Analysis and editing of manuscript.

## Declaration of competing interest

None declared.
